# Extracellular Acidic pH Inhibits Oligodendrocyte Precursor Viability, Migration, and Differentiation

**DOI:** 10.1371/journal.pone.0076048

**Published:** 2013-09-30

**Authors:** Anna Jagielska, Kristen D. Wilhite, Krystyn J. Van Vliet

**Affiliations:** 1 Department of Materials Science and Engineering, Massachusetts Institute of Technology, Cambridge, Massachusetts, United States of America; 2 Department of Biological Engineering, Massachusetts Institute of Technology, Cambridge, Massachusetts, United States of America; Hannover Medical School, Germany

## Abstract

Axon remyelination in the central nervous system requires oligodendrocytes that produce myelin. Failure of this repair process is characteristic of neurodegeneration in demyelinating diseases such as multiple sclerosis, and it remains unclear how the lesion microenvironment contributes to decreased remyelination potential of oligodendrocytes. Here, we show that acidic extracellular pH, which is characteristic of demyelinating lesions, decreases the migration, proliferation, and survival of oligodendrocyte precursor cells (OPCs), and reduces their differentiation into oligodendrocytes. Further, OPCs exhibit directional migration along pH gradients toward acidic pH. These *in vitro* findings support a possible *in vivo* scenario whereby pH gradients attract OPCs toward acidic lesions, but resulting reduction in OPC survival and motility in acid decreases progress toward demyelinated axons and is further compounded by decreased differentiation into myelin-producing oligodendrocytes. As these processes are integral to OPC response to nerve demyelination, our results suggest that lesion acidity could contribute to decreased remyelination.

## Introduction

Remyelination, a spontaneous regenerative process in the central nervous system (CNS), is considered a promising target of multiple sclerosis (MS) therapies, particularly in progressive phases for which current immunomodulatory treatments fail [1]–[5]. Remyelination has been demonstrated to prevent axon degeneration, the major pathological component of MS, and restore normal neurological function [6]–[12]. However, remyelination often fails in chronic stages of MS [13]–[16] for reasons not yet completely understood. Substantial effort is now directed toward improving our understanding of how the microenvironment of the MS lesion influences remyelination, to enable the development of effective therapies that promote myelin repair [2], [3], [17], [18].

The major cellular events after myelin loss that lead to remyelination are (1) the recruitment (proliferation and migration) of oligodendrocyte precursor cells (OPCs) to demyelinated axons; and (2) the subsequent differentiation of OPCs into myelinating oligodendrocytes that can regenerate myelin [18]. It is now recognized that these processes are regulated by multiple cell-dependent and microenvironment-dependent factors and can be affected by both biochemical and biomechanical pathological changes in MS lesion environment [2], [13]–[15], [18]–[33]. Among factors relatively less studied in the context of OPCs pathology, which are altered in demyelinating lesions compared to the healthy CNS, is the extracellular pH, which becomes acidic as a result of inflammatory processes and hypoxia [34]–[38]. Acidic pH has been recently measured in demyelinating lesion in the CNS of EAE mice (experimental autoimmune encephalopathy) as 6.60±0.23 versus 7.41±0.06 for healthy controls [36]. Because of the strong correlation between extracellular and intracellular pH in OPCs [39]–[42], and the effect of intracellular pH on multiple cell processes [36], [43]–[45] it is likely that extracellular pH may also affect OPC function. Moreover, we and others have shown the dependence of cell motility on pH in various cell types (bovine retinal endothelial cells [46], [47], human [48], [49] and mouse melanoma cells [50], breast cancer cells [51], and microglia [52]). This suggests that migration of OPCs in demyelinating acidic lesions could also be affected. However, the direct effect of acidic extracellular pH on OPC biology has not been yet demonstrated.

Here we show *in vitro* that migration of OPCs depends strongly on extracellular pH, decreasing with increasing acidity, and that this dependence is mediated in part by ligand-specific interactions between extracellular matrix (ECM) components and cell membrane. We further demonstrate that OPCs preferentially migrate toward acidic pH in pH gradients; such gradients are expected within demyelinating lesions to span the interface between healthy and demyelinated tissue. We also show that OPC proliferation, survival, and finally differentiation are decreased in an acidic environment *in vitro*. Based on these data, we propose that during post-demyelination recruitment of OPCs, the pH gradient may help to attract OPCs toward the acidic lesion from the surrounding healthy tissue. However, as the cells reach more acidic areas of a lesion, cell motility and attendant capacity to reach injured axons decreases; this is accompanied by a detrimental effect of the acidic environment on OPC proliferation and survival, and compounded by decreased differentiation potential. Together, these findings suggest that by affecting different components of OPC response to demyelination, acidic pH may be a contributing factor to the decreased remyelination potential of OPCs at lesion sites. Beyond the relevance to demyelinating diseases, these results may also have consequences in other biological contexts where OPCs are present, including brain tumor microenvironment and wound healing in brain injury; the documented ranges of extracellular pH are 6.2–6.9 for tumors [53]–[56] and 5.7–6.1 for wound healing [57].

## Methods

### Ethics Statement

Sprague Dawley rats were handled in the USDA-inspected MIT Animal Facility and all work involving the animals, including the primary use of these animals not presented in this study, and the spare rat cortex tissue harvesting and processing used in this study to obtain oligodendrocyte precursor cells, followed the guidelines from NIH and was approved by the Institutional Animal Care and Use Committee at the Massachusetts Institute of Technology (MIT Committee on Animal Care).

### Cell culture and media

OPCs were isolated from Sprague Dawley rat mixed glial cultures, as described previously [58]. Briefly, mixed glial cultures established from neonatal cortices were maintained in 10% fetal bovine serum (FBS) for 10–14 days prior to overnight shaking to remove OPCs. OPCs were maintained in a progenitor state in DMEM (Invitrogen) with SATO's modification (5 µg/ml insulin, 50 µg/ml holo-transferrin, 5 ng/ml sodium selenate, 16.1 µg/ml putrescine, 6.2 ng/ml progesterone, 0.1 mg/ml bovine serum albumin (BSA)) plus 10 ng/ml PDGF-A and 10 ng/ml FGF2 (Peprotech) (progenitor medium). To induce differentiation, OPCs were cultured in SATO's medium without FGF2 and PDGF-A and with 0.5% FBS (differentiation medium). To control the pH values during cell migration, survival, proliferation, and differentiation assays, a bicarbonate-free version of the cell progenitor or differentiation media described above was used (to eliminate pH sensitivity to CO_2_), with pH adjusted to a desired value using NaOH/HCl and pH meter with glass electrode. Experiments were conducted in room atmosphere, at 37°C. Before each experiment, cells were incubated in bicarbonate-free pH-specific media for 15 min, to allow for equilibration of cell-contained bicarbonate levels with media, and then media was refreshed. During the course of experiments, pH of the media was verified using colorimetric tests and was stable. Bicarbonate-free media has been used previously for many cell types in studies involving pH control [49], [52], [59]–[62], and we did not observe significant differences between experiments performed in bicarbonate-free media versus media containing bicarbonate, tested for migration at pH 7.4.

### Functionalization of glass-bottom and polystyrene dishes

#### Poly-D-lysine

Tissue culture polystyrene (PS; BD Falcon) and glass-bottom dishes (Invitro Scientific) were incubated for 1 h at 37°C with 5 µg/ml (PS) or 50 µg/ml (glass) poly-D-lysine (PDL, 70 kDa, Sigma), and washed twice with deionized water before cell seeding.

#### Laminin

Glass-bottom dishes were first incubated with 50 µg/mL PDL for 1 h at 37°C, and then washed twice with deionized water. The PDL-coated dishes were then incubated with laminin (mouse natural laminin-1, Invitrogen) for 1 h at 37°C, at different concentrations depending on the experiment (10–200 µg/mL), then washed twice with phosphate buffered saline at pH 7.4 (PBS; Gibco).

#### Fibronectin

Glass-bottom dishes were incubated with 10 µg/ml fibronectin (bovine plasma, Invitrogen) for 1 h at 37°C, then washed twice with PBS.

### Immunocytochemistry

The primary antibodies used for immunocytochemistry were rat anti-MBP (Serotec) used to measure OPC differentiation, rabbit anti-Ki67 (Millipore) used to measure OPC proliferation, and mouse anti-integrin α_6_β_1_ (Millipore) used to measure integrin expression in cells by flow cytometry. Secondary antibodies included goat anti-mouse IgM Alexa Fluor 488 (Invitrogen), goat anti-rabbit IgG Alexa Fluor 488 (Invitrogen), and rabbit anti-rat IgG Alexa Fluor 488 (Invitrogen).

### Adhesion, survival, proliferation, and differentiation assays

**Cell adhesion** to laminin-coated glass surfaces (10 µg/ml) at different pH levels was evaluated by the number of cells that attached to the surface from the cell suspension (130,000 cells suspended in 1.5 ml of pH defined-media placed in the 2 cm diameter, laminin-coated glass bottom well), after 1 h incubation in 37°C in pH-specific progenitor media on an orbital shaker rotating at 1 Hz frequency. After incubation, media with remaining suspended cells were removed, the attached cells were fixed with 4% paraformaldehyde and stained with Hoechst 33342 (Invitrogen). Stained nuclei were imaged with fluorescence microscope, and summed for 20 areas on the dish. This sum was averaged over three independent experiments and presented as percentage of the average cell number adhered at pH 7.0.

**Cell survival** was evaluated after 24 h incubation at 37°C in pH-specific progenitor media using live-staining with propidium iodide (PI, Invitrogen). Live cells were incubated with PI (5 µg/ml in progenitor media) for 15 min at 37°C, followed by three washes with PBS, fixing with 4% paraformaldehyde, and staining nuclei with Hoechst 33342 for counting of total number of cells (final concentration 2 µg/ml for 5 min). Stained OPCs were imaged using fluorescence microscope (Olympus IX-81), and survival was calculated as percentage of live cells (cells that did not stain red with propidium iodide) relative to a total number of cells (counted as number of nuclei stained with Hoechst), averaged over ten areas in the dish per experiment, imaged with fluorescence microscope, for three (pH 6.0 and 6.5) or two (pH 7.0, 7.5, and 8.0) experiments.

**Cell proliferation** was evaluated after 24 h incubation at 37°C in pH-specific progenitor media, by immunostaining against Ki67 protein and presented as percentage of fluorescent cells (Ki67 positive) with respect to a total number of cells, averaged over 20 areas per experiment imaged with fluorescence microscope, for six (pH 6.0 and 6.5) or four (pH 7.0, 7.5, and 8.0) experiments. After incubation, OPCs were first live-stained with PI to mark live cells, followed by three washes in PBS, fixing with 4% paraformaldehyde, immunostaining against Ki67 (performed at pH 7.4) followed by staining with secondary antibody with Alexa Fluor 488 (Invitrogen), and staining nuclei with Hoechst. Only cells that were living before immunostaining (did not stained red with PI) were considered.

**Differentiation** was evaluated after 5 days incubation in pH-specific differentiating media, by immunostaning against myelin basic protein, MBP, and presented as percentage of fluorescent cells (MBP positive) with respect to a total number of cells averaged over twenty areas per experiment, imaged with fluorescence microscope, for six (pH 6.0 and 6.5) or four (pH 7.0, 7.5, and 8.0) experiments. After incubation, cells were first live-stained with PI to mark live cells, followed by three washes in PBS, fixing with 4% paraformaldehyde, immunostaining against MBP followed by staining with secondary antibody with Alexa Fluor 488 (Invitrogen), and staining nuclei with Hoechst. Only cells that were living before immunostaining were considered.

#### Immunostaining

Cells were fixed with 4% paraformaldehyde for 20 min, washed with PBS, blocked with 1% BSA (bovine serum albumin) in PBS (blocking solution) for 30 min, and permeabilized with 0.1% Triton X100 for 3 min. Primary antibodies were diluted in blocking solution and incubated with cells at room temperature for 1 h. After three washes with PBS, cells were incubated for 1 h with secondary antibodies (diluted in PBS 1∶500, to final concentration 4 µg/ml). After three washes with PBS, cell nuclei were stained with Hoechst.

#### Flow Cytometry

Flow cytometry (BD LSR Fortessa) of fluorescently stained OPCs was used to compare expression levels of integrin α_6_β_1_ in OPCs incubated in media with different pH. Live OPCs adhered to laminin-coated surfaces (10 µg/ml) were incubated for 3 h at 37°C in progenitor media at different, fixed pH levels. After incubation, OPCs were detached with trypsin/EDTA, fixed in suspension with 4% paraformaldehyde for 10 min, and then washed twice by centrifugation in blocking solution (1% BSA in PBS). Cells were next incubated in blocking solution for additional 10 min, and immunostained with anti-integrin α_6_β_1_ primary antibody for 1 h (1∶20 dilution). After washing twice by centrifugation in blocking solution, cells were incubated for 1 h with secondary antibody tagged with Alexa Fluor 488, and then washed twice by centrifugation in blocking solution. Whole cell fluorescence was measured for three experiments per pH condition, each experiment done in triplicate. For each experiment, geometric mean fluorescence intensity was averaged for three samples per pH condition and expressed as a fraction of average value obtained for pH 7.0. These fractional fluorescence intensities were then averaged for three experiments. Unstained OPCs were used as a negative control, and OPCs stained only with secondary antibody were used to subtract non-specific fluorescence background.

#### Cell migration measurements in media with uniform pH

Migration parameters were calculated from cell migration pathways recorded over 4 h by time-lapse microscopy imaging with phase contrast (Olympus, IX-81, 10× magnification), with 3 min intervals between snapshots. Cells were plated in 60 mm-diameter, glass bottom dishes (30 mm glass diameter) coated with laminin, fibronectin, or PDL at the concentrations indicated for each experiment, and measured in bicarbonate-free progenitor medium (with growth factors) with pH adjusted to a desired value, at 37°C. Imaging commenced after 30 min of cell equilibration. For each pH condition, 30 OPCs per experiment were measured at different locations of the dish, experiments were repeated twice, and results were averaged over the entire set of sixty cells. From these pathways, cell migration velocity (calculated as an average over all 3 min-interval velocities), and radius of migration (maximum distance traveled from the starting location) were calculated using ImageJ software [63] with the module ‘Analyze Particles’ to determine coordinates of the cell centroid in each snapshot. Customized scripts were written to obtain migration velocity and radius.

#### Cell migration measurements in pH gradients

Migration of OPCs in a pH gradient was studied in a Zigmond chamber [64]. OPCs were grown on laminin-coated cover glass (10 µg/ml, functionalized at pH 7.4), which was placed on top of the chamber wells divided by a bridge of 1 mm width; OPCs faced the chamber. The left well was then filled with 100 µL of progenitor media (containing FGF2 and PDGF-A growth factors) with pH 6.0, and the right well with 100 µL of progenitor media with pH 7.0. The two solutions interfaced in the volume above the 1 mm bridge where the pH gradient was formed. The space between the bridge and cover glass with OPCs was adjusted to 50 µm with Teflon spacers, to allow for unconstrained OPC migration while maintaining stable pH gradients. For control experiments with no pH gradient, both wells were filled with progenitor media with pH 7.0. To avoid media evaporation and air intake, the edges of the cover glass contacting the chamber were sealed with wax mix (1∶1∶1 w/w mixture of beeswax, paraffin, and vaseline). The pH gradient stability during the 4 h imaging time was confirmed in a separate experiment with BCECF (10 µM) pH sensitive dye (2′,7′-bis-(2-carboxyethyl)-5-(and-6)-carboxyfluorescein, Invitrogen). The above-assembled Zigmond chamber was mounted on inverted optical microscope that was incubated at 37°C (Olympus IX-81), and equilibrated for 30 min before time-lapse imaging to allow for pH gradient stabilization. Migration velocities, radius of migration, and percentages of cells migrating toward or away from acidic well in pH gradient and control experiments were calculated from 4 h cell migration pathways, as described in “Cell Migration Measurements in media with uniform pH.” The orientation of the pH gradient (i.e., a line parallel to the shorter bridge axis) was aligned with the x-coordinate in the microscope images, and migration was defined as “toward acidic region” (or left well, for control experiments) when the change of a cell's x-coordinate was negative, and as “away from acidic region” (or right well, for control experiments) when the change of a cell's x-coordinate was positive. For each cell, the initial x-coordinate was assigned to 0, regardless of cell position in the bridge.

#### Cell stiffness measurements

Cell stiffness (effective Young's elastic modulus *E*) was measured at pH 6.0 and 7.0 using atomic force microscope (AFM)-enabled nanoindentation (MFP-3D Bio AFM, Asylum Research). A silicone nitride cantilever with an attached polystyrene bead of 25 µm diameter and a nominal spring constant *k* = 0.03 N/m (Novascan) was employed; the actual spring constant was calibrated via the thermal noise method [65]. *E* was measured for cells adhered to PDL-coated dish and incubated at 37°C in media with pH 6.0 or 7.0 (15 cells per pH condition). Ten force-indentation curves were collected for each cell at the cell body center and fitted to the Hertzian model [66] for an indentation depth of 0.4 µm, to obtain *E*. Mean cell stiffness was reported for each condition.

#### Optical microscopy image acquisition

For cell migration image acquisition (Olympus IX-81 with Orca-R2 camera and Prior motorized stage), optical phase contrast images were acquired at 10× magnification (Olympus UPlanFLN 10×, N.A. 0.30) using Metamorph imaging software, at 37°C in pH defined cell specific media (see “Cell culture and media”). Migration data was analyzed using ImageJ software. For cell survival, proliferation, and differentiation assays, images were acquired (Olympus IX-81 fluorescent microscope, equipped with Lumen fluorescent lamp and Orca2 camera) at 10× magnification (Olympus UPlanFLN 10×, N.A. 0.30) at room temperature in PBS. Cells were stained using propidium iodide (PI), fixed with 4% paraformaldehyde, and stained with primary antibodies followed by secondary antibodies with Alexa Fluor 488 (Invitrogen).

#### Statistical analysis of data

Reported errors were standard errors of the mean, SEM. Statistical significance analysis was conducted by one way ANOVA followed by Bonferroni tests.

## Results

### Migration of OPCs on laminin and fibronectin surfaces decreases in acidic pH

**Figure 1:**
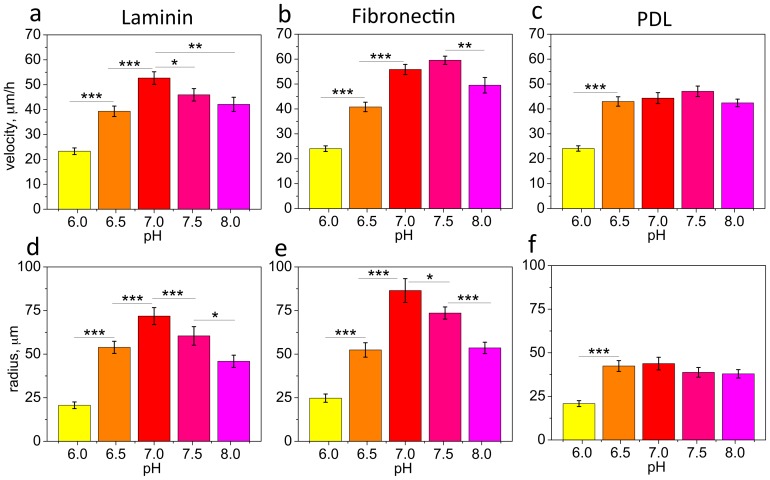
Migration velocity and migration radius of OPCs decreases at acidic pH. (**a, d**) laminin (10 µg/ml), (**b, e**) fibronectin (10 µg/ml), and (**c, f**) PDL surfaces (50 µg/ml). Shown are mean values for N = 60 cells per pH condition. (**a–f**) Error bars are SEM; * p<0.05, ** p<0.01, *** p<0.001. Colors correspond to cell media pH, representing schematically the colors on a pH indicator strip.

Motivated by recent results confirming acidic pH in demyelinating lesions [36] and recent findings demonstrating the dependence of cell migration on extracellular pH (pH_e_) for CHO-B2 and vascular endothelial cells [46], [47], we investigated how acidic pH may affect migration of OPCs. *In vivo*, in response to demyelination, OPCs are recruited to the predominantly acidic lesion and thus migrate in an environment characterized by changing pH. We conducted time-lapse imaging of OPC migration over 4 h at 37°C, in cell media covering a range of uniform pH from 6.0 to 8.0, which spans the pH range reported for a healthy and lesioned neural tissue (6.3–7.4, [36]). Migration of OPCs was analyzed on surfaces coated with laminin, fibronectin, or poly-D-lysine (PDL); see Methods. Laminin is a major component of the ECM in the central nervous system [67]. Fibronectin content in the ECM of the CNS is relatively lower [67], but is increased in lesioned neural tissue [27], [68]. OPCs interact with laminin and fibronectin through membrane proteins, including integrins α_6_β_1_ - a receptor for laminin, and the receptors for fibronectin: α_v_β_1_ and α_v_β_3_ (α_v_β_3_ expressed at lower levels at oligodendrocyte progenitor stage) [69], [70]. A PDL-coated surface was used as a control to evaluate OPC migration as a function of pH_e_ in the absence of specific binding ligands (i.e., adsorbed ECM proteins) between the cell membrane and migration surface. We observed a biphasic dependence of OPC migration velocity and migration radius (the largest distance the cell traveled from the initial position) on extracellular pH, for laminin and fibronectin surfaces, with maxima at pH 7.0 (on laminin) or 7.5 (on fibronectin) (Figs. 1 a, b, d, e). In acidic pH (6.0 and 6.5), both the migration velocity and migration radius decreased significantly (Figs. 1 a–b, d–e). In contrast, on surfaces coated with PDL that does not specifically bind to cell membrane proteins, no such dependence was observed over the range of pH 6.5–8.0 (Fig. 1 c, f); migration velocity and radius decreased at pH 6.0. Overall, these data suggest that specific interactions between cell membrane proteins and ECM ligands are required for a response of cell migration to change of pH over the range of pH 6.5–8.0. The exception of pH 6.0, for which migration decreased on all tested surfaces including PDL, suggests that additional cell changes may have been triggered at this extreme pH beyond the specific ligand-cell surface receptor interactions.

### OPCs preferentially migrate toward acidic pH in pH gradient

The existence of an acidic MS lesion in the vicinity of healthy tissue (pH ∼7.4) plausibly creates a pH gradient to which OPCs are exposed during migration. Such gradients have been documented in other pathological contexts including tumor interior [54], [55], the interface between tumor and healthy tissue [71], and in wound healing environment [72]–[74]. We next asked how OPC migration is affected in a pH gradient. Although actual *in vivo* pH gradients in the MS lesion area have not been reported to date, these can be approximated grossly from a measured pH range (in mouse spinal cord: ∼6.60 (0.23) vs. 7.41 (0.06), for lesioned and healthy tissue, respectively, SEM in parenthesis [36]) and approximate lesion widths of sub-mm to a few mm [75].

**Figure 2:**
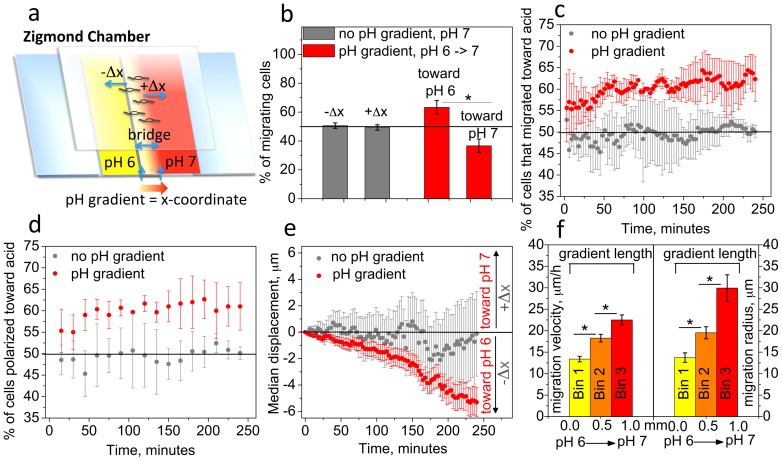
OPCs preferentially migrate toward acidic pH in a pH gradient. (**a**) Zigmond chamber schematic: for pH gradient, left and right wells filled with cell media at pH 6.0 (yellow) and pH 7.0 (red), respectively; for controls (no pH gradient) both wells filled with pH 7.0 media. X-coordinate aligned with pH gradient direction (shaded arrow) over 1 mm bridge. OPC displacement on laminin-coated cover glass toward acidic (or left) well corresponds to −Δx. Migration monitored for 4 h at 37°C. (**b–e**) Red: pH gradient; gray: control; (**b**) Percentage of cells shifted toward acidic (−Δx) or neutral (+Δx) well, with respect to cell initial x-coordinate. (**c**) The same cell percentage as in (b) calculated for each time point with 3 min interval, over 4 h observation. (**d**) Percentage of cells polarized toward acidic well (left, −Δx) with respect to the cell position at the previous time point, calculated with 15 min interval. (**e**) Median displacement along x-coordinate for pH gradient (red) and control (gray) conditions. (**f**) Mean migration velocity and migration radius calculated in three bins evenly spaced along the pH gradient (N = 50 cells per bin). Colors represent different pH ranges within each bin, from more acidic in bin 1 to less acidic in bin 3. For (**b–d**), each data point is mean from three experiments, with N = 100 cells per experiment; for (**e**), each data point is median displacement calculated for the all cells from three experiments (N = 300 cells). (**b–f**) Error bars are SEM; * p<0.05, ** p<0.01, *** p<0.001.

Our further investigations of OPC migration were focused on laminin surfaces - the major component of ECM in the CNS. We used a Zigmond chamber (Fig. 2a) to create a gradient spanning over 1 mm from pH 6.0 to 7.0. The distance of 1 mm over which the pH gradient is created spans the range of observed diameters of MS lesions [75], and is also within a typical recruitment radius of OPCs to the lesion (∼2 mm) [76]. We chose the pH range from 6.0 to 7.0 that corresponded to the observed OPC velocity reduction on laminin (from 7.0 to 6.0, Fig. 1a) and also included the pH gradient relevant to MS lesions. Here, the left well of the Zigmond chamber was filled with media at pH 6.0 and the right well with media at pH 7.0. OPCs were grown on cover glass coated with laminin (10 µg/ml), which was placed above the wells such that OPCs were in contact with media in the wells and above the bridge (Fig. 2a). A pH gradient spanning from pH 6.0 to 7.0 was formed at the interface of the two solutions that intersected in the narrow space above the 1 mm wide bridge, over which OPC migration was imaged. Control experiments were conducted with no pH gradient, with both wells filled with media at pH 7.0.

In migration analysis, we considered cell movement along x-coordinate parallel to a short axis of the bridge, which aligned with the pH gradient (Fig. 2a, shaded arrow). Negative changes in x-coordinate corresponded to cell movement toward the acidic region (away from pH 7.0; −Δx in Fig. 2a). Time-lapse imaging of OPCs over 4 h at 37°C revealed that OPCs migrated preferentially toward the acidic region. Figure 2b shows that 63% of cells migrated toward the acidic region within 4 h, with respect to their initial position in the beginning of the experiment. In control experiments lacking a pH gradient, there was no significant difference in the percentage of cells that migrated toward the left (−Δx) or right (+Δx) well. Figure 2c shows how the percentage of cells that migrated toward acid changed with time. In the pH gradient, at any time point more cells moved toward the acidic well; this trend increased from 55% at t = 3 min to 63% at t = 4 h. (Note that imaging of migration commenced after a 30 min equilibration period, which may explain the observed directionality of migration already at *t* = 3 min). This increase in the fraction of OPCs that migrated toward acid over time indicates that cells gradually polarized (turned) toward the acidic end of pH gradient [47]. Control conditions resulted in fluctuations of cell fraction at ∼50% during the course of the experiment, indicating no preference of OPC migration direction in the absence of a pH gradient (Fig. 2c).

In Fig. 2c, the number of cells that migrated toward acid in the pH gradient is calculated based on an x-coordinate shift with respect to the initial coordinate of each cell; therefore, this shift does not explain whether cells systematically migrated toward acid at all time points during the course of experiment, or whether there was only initial directional migration toward acidic well followed by predominantly non-directional cell movements. To clarify this point, we analyzed how many cells were actively migrating toward the more acidic region at each 15 min interval over 4 h (termed “polarization toward acid”, [47]). Here, migration was considered with respect to the x-coordinate at the previous time point (15 min earlier). Figure 2d shows that, in the pH gradient, at any time point more OPCs are polarized toward the acidic well, increasing from 55% at t = 15 min to 61% at t = 4 h; this indicates a persistent migration of the cell population toward acid. When no pH gradient was present, fluctuations hovered at 50%, showing no preference of OPCs migration in any direction at any time point. Figure 2e shows median cell displacement with respect to initial cell position for each time point, with 3 min intervals. In this pH gradient, there is a systematic change of the cell population median x-coordinate toward acidic pH; the control indicated no significant shift of the population, and the deviation from zero at t = 175 min was not statistically significant. The lower SEM for median displacement in the pH gradient (Fig. 2e, red) indicated a narrower distribution of cell displacements, and therefore more uniform migration of the OPC population compared to no gradient condition, at pH 7.0. Figure 2f shows mean cell velocity and migration radius in three bins evenly spaced along the pH gradient (bin 1 closest to the well at pH 6.0 and bin 3 closest to the well at pH 7.0), averaged for 50 cells per bin. Migration velocity and radius were lowest for cells located in the most acidic region of the gradient (bin 1); this is consistent with cell migration dependence on uniform pH (Fig. 1). (Note that mean migration velocity and radius was generally lower in the pH gradient (Fig. 2f) as compared to the uniform pH (Fig. 1a). This may be attributable to slight differences in experimental setup including the migration volume and adsorbed ligand density.)

### OPC adhesion and length increase in acidic pH

**Figure 3:**
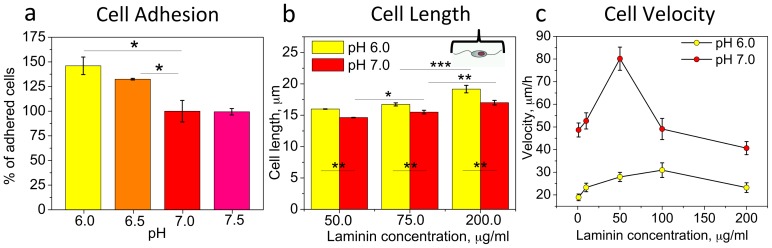
OPC adhesion and length increase at acidic pH. (**a**) Cell adhesion to laminin-coated glass surfaces (10 µg/ml) at different media pH was evaluated as the percentage of cells attached after 1 h incubation in 37°C on orbital shaker rotating with 1 Hz frequency. Data are mean from three experiments relative to percentage of cells adhered at pH 7.0. (**b**) Dependence of OPC length on extracellular pH (for pH 6.0 and 7.0), for different laminin coating concentrations. Cell length is calculated as a distance between the ends of two longest cell processes (schematically shown in the right top corner). Data are mean from two experiments per condition, N = 50 cells per experiment. (**c**) Dependence of OPC migration velocity on laminin coating concentration, for pH 6.0 and 7.0. Data are mean for N = 60 cells per point. For each laminin concentration, the difference between cell velocity at pH 6.0 and 7.0 is statistically significant. (**a–c**) Error bars are SEM; * p<0.05, ** p<0.01, *** p<0.001. Colors correspond to cell media pH.

Cell migration requires reversible adhesion to the underlying surface, mediated by interactions with surface ligands [77]–[79]. We next examined how adhesion of OPCs to laminin-functionalized surfaces depended on pH_e_. Figure 3a shows cell adhesion at different, uniform pH conditions after 1 h incubation, expressed as percentage of cells that attached to the surface relative to that in pH 7.0. OPC adhesion to laminin increased with increasing acidity of the media, which was correlative with slower migration of OPCs in acidic pH. These adhesion results were in agreement with analysis of cell length, calculated as a distance between the endpoints of opposing cell processes of an adherent OPC (see Fig. 3b, schematic). For OPCs, cell length is an indicator of cell spreading, as these cells interact with a surface by extending or contracting processes, with no signification changes in the spread area of the cell body. Cell length was larger at pH 6.0 as compared to pH 7.0, and increased with concentration of laminin for both pH conditions. Mean migration velocity as a function of laminin concentration for pH 6.0 and 7.0 (Fig. 3c) exhibited biphasic behavior, as is consistent with many migrating cell types [46], [80]. Note that at any laminin concentration, OPC velocity at pH 6.0 was always lower than that at pH 7.0.

**Figure 4:**
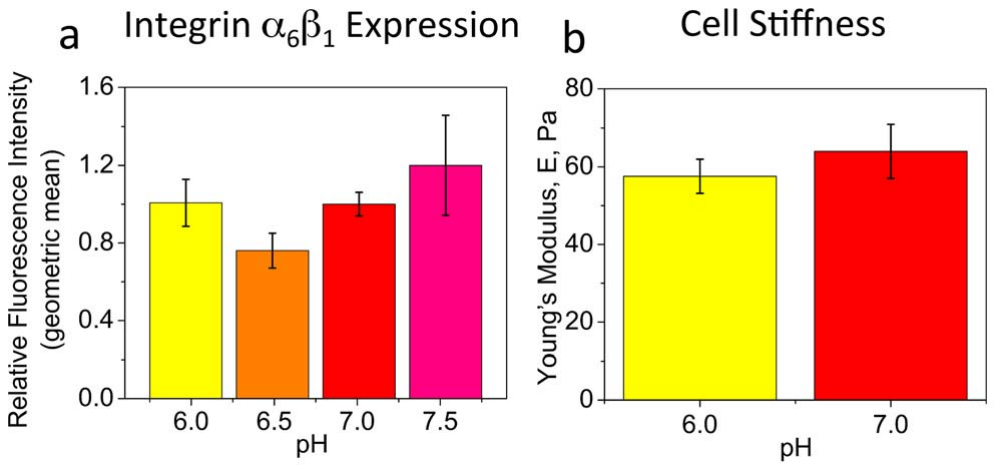
Expression of integrin α_6_β_1_ and stiffness of OPCs at different pH. (**a**) Expression level of integrin α_6_β_1_ at different extracellular pH, evaluated by OPC immunostaining against α_6_β_1_ (with Alexa Fluor-488 fluorochrome) and analysis of cell fluorescence using flow cytometry (BD LSR Fortessa). Data are geometric mean fluorescence intensities averaged over three experiments, each conducted in triplicate, and presented relative to value obtained for pH 7.0. No statistical difference was observed between any pH conditions. (**b**) Cell stiffness at pH 6.0 and 7.0, evaluated using AFM-enabled nanoindentation. Data are mean of Young's elastic modulus measured for 15 cells per condition. No statistical difference was observed between pH 6.0 and 7.0. Error bars are SEM. Colors correspond to cell media pH.

As the OPCs exhibited increased cell adhesion to laminin with decreased pH_e_, we also investigated the possible involvement of integrin α_6_β_1_, the major laminin receptor, in mediating the response of OPC motility to pH_e_. Analysis of expression levels of integrin α_6_β_1_ in OPCs incubated for 3 h in pH-specific media (time scale similar to migration experiments) on laminin (10 µg/ml), evaluated with whole cell immunostaining followed by flow cytometry, did not indicate statistically significant differences between different pHs (Fig. 4a). Attempts to measure dissociation constants for the laminin-integrin complex at different pH via surface plasmon resonance (SPR, Biacore 2000) were inconclusive. Therefore, at present we can exclude differences in integrin α_6_β_1_ expression as the mechanism of the pH-dependent OPC migration response on laminin, but cannot rule out potential differences in integrin binding affinity; see Discussion. It is also unlikely that pH induces major conformational changes in laminin, as no significant structural changes in laminin were shown at wide range of pH (4.0–7.4) [81], [82]. To ensure even ligand surface density in pH experiments, surface functionalization (for all ligands) was conducted at pH 7.4, prior to migration experiments in pH-altered media. Further, although it is predicted that cell stiffness can modulate migration velocity [47], [80], we measured no significant differences in effective Young's elastic modulus of OPCs at pH 6.0 and 7.0, via atomic force microscopy (AFM)-enabled nanoindentation (Fig. 4b).

### OPC survival, proliferation, and differentiation are decreased in acidic extracellular pH

**Figure 5:**
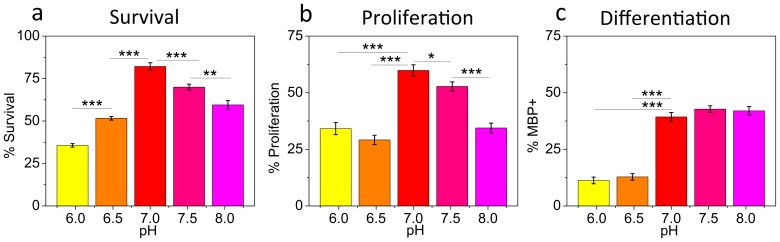
OPC survival, proliferation, and differentiation decrease in acidic extracellular pH (PDL surface, 50 µg/ml). (**a**) Survival was evaluated as percentage of live cells (detected with PI staining) relative to a total number of cells (detected via Hoechst staining). Data are from three (pH 6.0 and 6.5) or two (pH 7.0, 7.5, and 8.0) experiments. (**b**) Proliferation was evaluated by immunostaining against Ki67 protein and expressed as percentage of Ki67 positive cells with respect to a total number of cells. Data are mean for six (pH 6.0 and 6.5) or four (pH 7.0, 7.5, and 8.0) experiments. (**c**) Differentiation evaluated by immunostaning against myelin basic protein, MBP, and expressed as percentage of MBP-positive cells with respect to a total number of cells. Data are average for six (pH 6.0 and 6.5) or four (pH 7.0, 7.5, and 8.0) experiments. (**a–c**) Error bars are SEM; * p<0.05, ** p<0.01, *** p<0.001. Colors correspond to cell media pH.

Remyelination requires not only migration toward a demyelinating lesion, but also OPC survival, proliferation, and differentiation into myelin-producing oligodendrocytes. Thus, we next examined the influence of pH_e_ on these processes (Fig. 5). Here, we focused on pH effects independent of ligand-binding at the cell-surface interface, to allow for direct comparison with other published results obtained for cells on biologically inert surfaces [39], [83], and conducted these experiments on PDL-coated surfaces, to exclude possible compounding effects of integrin-ECM binding on OPC survival, proliferation, and differentiation [84]–[87]. We observed that OPC survival (evaluated by propidium iodide staining, Fig. 5a), proliferation (evaluated by immunostaining against Ki67 protein; Fig. 5b), and differentiation (evaluated with immunostaining against myelin basic protein, MBP; Fig. 5c) all decreased in acidic pH as compared to more physiological pH 7.0–7.5. Survival and proliferation were maximal at pH 7.0, whereas differentiation was independent of pH for pH≥7.0.

## Discussion

Remyelination can prevent axon deterioration and restore neurological function in demyelinating diseases including multiple sclerosis [6]–[12]; it is considered among the most promising therapeutic avenues in progressive MS. *In vivo*, this regenerative process requires oligodendrocyte precursors to migrate, proliferate, survive, and ultimately differentiate and remyelinate axons, and it often fails in chronic MS due to the pathological lesion microenvironment that reduces remyelination potential of oligodendrocytes [2], [17], [18]. Although multiple biochemical factors [1], [2], [16]–[19], [24], [29]–[33], [88]–[91] and biomechanical conditions [25]–[27] have been identified in MS lesions that contribute to failure or enhancement of remyelination, our knowledge of this pathological environment remains incomplete. Here, we focused on the influence of acidic extracellular pH on OPC biology, a relatively less studied factor present in demyelinating lesions [36], and demonstrated that acidic pH decreased OPC migration, proliferation, survival, and differentiation to myelinating oligodendrocytes. We also showed that OPCs preferentially migrated toward acidic pH, over a pH gradient that is plausibly representative of that in demyelinating lesions. Although the detailed mechanisms regulating influence of extracellular pH on these complex processes are beyond the scope of the current study, the consideration of these first *in vitro* findings in context of previous studies and of *in vivo* implications may prompt future explorations of correlation and causation.

### pH gradients may enhance recruitment of OPCs to demyelinating lesions

We observed that OPCs migrated predominantly in the direction of acidic pH within a gradient (Fig.2). Although detailed measurements of pH gradient profiles in demyelinating lesions have not yet been reported, this *in vitro* gradient range is plausible *in vivo*. Specifically, the pH of lesioned CNS tissue (pH 6.6 (0.23) for EAE mice [36] and 6.2 for ischemic/hypoxic conditions [92]–[97]) is distinct from ostensibly adjacent healthy CNS tissue (pH 7.4 (0.04)) [36]. The minimum and maximum pH values in the gradient range used here corresponded to the largest difference in average cell velocity that we observed on laminin surfaces (Fig. 1a), providing the opportunity to observe directional pH-dependent migration. The pH gradient distance *in vitro* (1 mm) was within a range of observed MS lesion diameters [75], and a typical recruitment radius of OPCs to the lesion (∼2 mm radius around the lesion [76]).

OPC migration toward the more acidic region of the gradient was persistent through the duration of the experiments, and cells apparently polarized so that the OPC population gradually shifted toward the acidic region (Fig. 2). This suggests that *in vivo* pH gradients at the lesion/healthy tissue interface may promote OPCs recruitment toward acidic lesions. The *in vivo* mechanism of OPC recruitment to demyelinating lesions is less understood, compared to developmental migration of OPCs [18], [98], [99]. Although multiple potential biochemical attractants have been indicated at lesions [18], [21], [29], [33], [100]–[109], it is unknown which factors dominate in OPC recruitment and whether pH gradients may be additional significant factors *in vivo*. However, our migration studies were carried out in the presence of constant physiological concentrations of two known biochemical lesion attractants, PDGF-A and FGF-2 (10 ng/ml), and indicated a significant effect of pH gradient on OPC migration directionality. Cell velocity decreased with increasing proximity to the acidic zone (Fig. 2f), indicating that as OPCs migrate toward acidic regions the cells gradually slow down. A speculative but possible implication for OPC recruitment to demyelinating lesions could be that, as OPCs migrate toward an acidic lesion in a pH gradient, the progenitor cells are kinetically arrested in more acidic areas of a lesion due to decreased motility; this reduced velocity would also reduce likelihood of finding and interacting with demyelinated axons.

Directional OPC migration toward the acidic zone is consistent with an earlier study for endothelial cells and CHO-B2 cells expressing α_v_β_3_ [47]. In that study on fibronectin surfaces, predominant polarization of cells toward acid was identified as a major mechanism of directional movement toward acid. Cell polarization was further correlated with increased lifetime of cell protrusions, stabilized by the increased number of actin-integrin adhesion complexes in acidic media [47]. In the current study, we observed similar polarization of OPCs toward acid (Fig. 2d), which can be rationalized by increased general cell adhesion to the surface ligands in more acidic conditions (Fig. 3a). This also agrees with earlier observations of directional cell migration in a ligand density gradient, toward regions of higher fibronectin density [110], [111] corresponding to the increased cell-surface adhesion. These consistent findings for different cell types suggests that directional cell migration toward acidic pH may be a more general phenomenon that facilitates cell recruitment to pathological microenvironments with inflammatory conditions characteristic of lower pH – including wounds, tumors, and inflammatory demyelinating lesions.

In contrast to our observations for OPCs, Faff et al. [52] reported lower numbers of microglia migrating from a neutral-pH to an acidic well in a Boyden chamber, compared to migration between uniformly neutral-pH wells. However, it is difficult to interpret that experiment in terms of migration along a pH gradient, because of the membrane-constrained migration through 8 µm pores and a short migration length limited to membrane thickness of 6–10 µm (comparable to cell size). Migration experiments within a Zigmond chamber, as used here, allow for unconstrained cell migration in pH gradients of extent and length that more closely approximate those expected *in vivo*.

### Effect of extracellular pH on OPC migration is mediated by ECM ligand-cell surface receptor adhesion

Reduced OPC motility was observed in response to acidic extracellular pH on both laminin and fibronectin (Figs. 1a–b, d–e), at a given ligand concentration. OPC motility was also reduced at the increased ligand density with ostensibly greater cell-ligand adhesion (Fig. 3c, for both pH 6.0 and 7.0 on laminin). This suggests that pH response may be facilitated by altered OPC adhesion to surface ligands. Indeed, direct measurements at different uniform pH levels showed increased OPC adhesion in acidic pH (Fig. 3a). These results correlated well with increased cell length at lower pH (compared were pH 6.0 and 7.0), and at higher laminin density (Fig. 3b), further supporting the connection between pH and cell-matrix adhesion. The biphasic dependence of cell migration velocity on laminin concentration (Fig. 3c) is characteristic of predominantly adhesion-mediated migration [80], and pH-dependent adhesion and migration characteristics were observed previously for other cell types [48]–[52], [112].

Although the molecular mechanisms regulating cell adhesion and motility via extracellular pH are not fully understood and are beyond the scope of this work, this dynamic process involves multiple components [113], [114]. These include ligand-receptor interactions at the cell-surface interface [46], [115]–[118], intracellular signaling that regulates membrane receptor expression and cytoskeleton organization [52], [119], [120], and other intracellular processes that may be mediated by changes of intracellular pH and alterations of ion channels functioning in response to extracellular pH [49], [112], [121]–[124]. pH-induced change of integrin conformation has been demonstrated in our earlier study for the specific integrin α_v_β_3_ in CHO-B2 cells, and correlated with subsequent change in integrin-fibronectin binding; the expression of α_v_β_3_ integrin receptors was unchanged in different pH, and instead the effective on-rate of integrin-fibronectin binding increased due to the higher likelihood of conformational opening and activation of the integrin itself [46]. In agreement with that work, here we observed the dependence of OPC migration on extracellular pH on laminin and fibronectin surfaces, but not on the PDL (Fig. 1), for a pH_e_ range of 6.5–8.0; this suggests that, within this pH range, specific interactions between cell membrane receptors and extracellular matrix ligands are required to mediate migration response of OPCs to extracellular pH.

We investigated the possible involvement of integrin α_6_β_1_, a major OPC receptor for laminin that is the dominant CNS extracellular matrix component, in mediating OPC motility as a function of extracellular pH. The major factors that could affect the laminin-integrin interface, and therefore cell migration in response to extracellular pH change, are levels of integrin expression, significant changes in surface density or conformation of laminin, and altered integrin-laminin binding dynamics. We found no statistically significant differences between expression levels of this integrin at the tested range of pH (6.0–7.5), based on cell immunostaining against α_6_β_1_ followed by flow cytometry (Fig. 4a). Significant differences in ligand surface density were excluded, in that laminin surface functionalization was conducted at pH 7.4, prior to migration experiments in pH-altered media. It is also unlikely that pH induces major conformational changes in laminin, based on dynamic light scattering experiments that have shown no significant structural changes in laminin at wide range of pH (4.0–7.4) [81], [82]. However, it remains possible that pH change could alter protonation or conformation of the binding site and affect binding affinity of the integrin-laminin complex; this correlation can be explored in future studies.

Other membrane receptors expressed by OPCs that were linked to OPC migration in neutral pH, including fibronectin-binding integrins α_v_β_1_ and, expressed at lower levels, α_v_β_3_, and proteoglycans (e.g., heparan sulfate proteoglycans, chondroitin sulfate proteoglycans, α-dystroglycan) [70], [98], [125]–[127] may play a role in mediating pH-dependent OPC migration, and could be considered in future studies of associated mechanisms. Finally, other receptor-ligand independent factors, such as intracellular acidification or ion exchange alterations may possibly play a more significant role in OPC migration for pH<6.5. In summary, these results show that OPC migration over the extracellular pH range 6.5–7.5 includes specific ligand-receptor binding that influences cell-surface adhesion. Increased cell adhesion to laminin in acidic pH was not related to changed expression levels of α_6_β_1_, indicating that altered binding interaction details may be an important part of the response to extracellular acidity.

### Acidic pH may negatively affect OPC survival, proliferation, and differentiation in demyelinating lesions

We studied the effect of extracellular pH on OPC survival, proliferation, and differentiation on PDL surfaces (i.e., independent on specific ligand binding). Survival of OPCs was maximal for extracellular pH of 7.0, with lower survivability in more acidic and in more alkaline pH (Fig. 5a). Survivability decreased more dramatically in increased acidity. Although the effect of ischemic/hypoxic acidification on brain tissue damage has been studied extensively [35], [36], [43], [128]–[132], to our knowledge this is the first direct measurement of extracellular pH effects on OPC survival. A possible link between acidic environment and OPC death has been suggested by Feldman et al. [43], through involvement of acid-sensing channel-1a (ASIC1a) expressed in OPCs and increased Ca^+2^ influx. Extracellular alkaline pH effects on cell survival have been generally less studied than acidification effects [133]–[136]. However, in the CNS, transient alkalization may follow ischemic acidification events [136]; This alkaline-induced increase in cell death has also been reported for several cell types including human endothelial cells [133], in association with increased activation of caspase-3 pathway and subsequent apoptosis; and a murine fibrosarcoma cell line, in association with elevated Ca^+2^ and mitochondrial damage [135].

Proliferation of OPCs showed biphasic behavior as a function of extracellular pH (Fig. 5b) that was similar to that of OPC survival, with a maximum for pH_e_ 7.0. To our knowledge, this is the first report of direct extracellular pH effects on OPC proliferation. A similar biphasic proliferation profile in response to extracellular pH, with the maximum for pH_e_ ∼7.0, was observed by Pappas et al. [83] for astrocytes, for pH_e_ ranging 6.5–7.8, and was linked to the subsequent change of intracellular pH, pH_i_. As changes in pH_i_ can affect progression through S-phase of the mitotic cycle [137] and the activity of different potassium channels involved in cell proliferation [83], [138], [139] it is likely that proliferation dependence on pH_e_ in OPCs is also mediated through correlated changes of pH_i_, for which dependence on pH_e_ has been well documented [39]–[42]. A biphasic proliferation profile as a function of pH_i_ was also shown for fibroblasts [140] and kidney cells [141], [142]. These broad findings support the concept of a range of pH_e_/pH_i_ that is permissive for proliferation, below or above which proliferation is significantly decreased. Interestingly, Bousouff et al. [39] showed that steady state pH_i_ is more acidic for OPCs compared to differentiated oligodendrocytes (6.88 vs. 7.04, respectively), and suggested that intracellular alkalization beyond the proliferation permissive value during OPC differentiation may be a factor that inhibits proliferation in adult oligodendrocytes. This suggestion agrees well with our measurements of lower OPC proliferation in alkaline pH (Fig. 5b).

Differentiation of OPCs, quantified in terms of percentage of cells expressing MBP after 5 days in differentiating medium, decreased for pH≤6.5 (Fig. 5c). Analysis over this wider range of extracellular pH (6.0–8.0) included pathologically relevant acidic pH; no significant difference was identified for pH between 7.0 and 8.0. This reduced OPC differentiation in acidic pH suggests that the acidic conditions of demyelinating lesions could have a similar inhibitory effect. Although we identified no significant difference in the fraction of cells expressing MBP at extracellular pH 7.0–8.0, Bernard et al. noted differences in the percentage of cells expressing the GalC differentiation marker after 3 days in differentiating media [143], for this pH range. The expression of GalC was biphasic with a maximum for extracellular pH 7.8 (intracellular pH 7.15), and Bernard et al. considered pH to be a key regulator of OPC differentiation via activation of the ERK1/2 pathway.

### Physiological significance of acidic pH in remyelination

The significance of these observed effects of acidic pH on OPC biology *in vitro* is not yet clear in the context of *in vivo* demyelinating lesions, and prompts future mechanistic studies. Detailed measurements of spatial and temporal pH distributions in lesions, and their correlations with OPC proliferation, migration, differentiation, and remyelination, are not yet available for either human patients or animal models of demyelinating tissue. There is, however, clear evidence of acidic pH measured locally in the CNS of EAE mice [36], and a strong premise of acidosis in MS lesions based on the correlations of demyelination with levels of metabolites such as lactate or N-acetyl aspartate [34], [144]. Those characteristics are also indicated in ischemic/hypoxic conditions associated with acidic pH (∼6.2 [92]–[97]).

In the complex biochemical millieu of *in vivo* demyelination, pH plausibly acts together with multiple other cues, and outcomes for OPC response will depend on the relative levels of all contributing factors that vary with space and time/disease stage. Given the high cytological and biochemical heterogeneity among demyelinating lesions and within individual lesions [75], [145], and changes in both during disease progression, it is possible that in some lesion sites the negative modulation of OPC biology by acidic pH will be significant, and in other regions or stages the pH effect will be muted by stimulants of myelin regeneration. We note that our observed effects of pH on OPC biology *in vitro* occurred at physiological concentrations of PDGF-A and FGF2, potent mitogens and chemoattractants that are present in demyelinating lesions [100], [101]. Remyelination failure is usually attributed to insufficient OPC recruitment to the lesion or/and lack of OPC differentiation to myelinating oligodendrocytes [18]; both deficiencies can occur in the same individual [75], [145]–[147]. We showed that acidic pH *in vitro* is capable of affecting both OPC recruitment and differentiation. During initial stages of MS, OPCs typically rapidly respond to myelin loss with increased proliferation and migration toward the lesion, often followed by relatively efficient differentiation and remyelination [13], [18], [75], [145], [146]. Efficient OPC repopulation of newly formed (non-chronic) lesions has also been observed in animal models of experimental demyelination (both autoimmune and toxin-induced) and in response to physical CNS trauma (e.g., stab wounds) [18], [76], [108], [148]–[155]. OPC differentiation and remyelination in these experimental models has also been reported, particularly extensive for toxin-induced demyelination [18], [156], despite likely acidic lesion conditions [36]. Those findings indicate that, at initial MS stages and in related animal models, pro-regenerative factors dominate the lesion environment. However, even for a MS lesion generally described as remyelinating at the initial disease stage, there are differences in OPC densities and remyelination extent within the lesion [147]. This diversity of response could be due to heterogeneous distribution of biochemical factors [147], which may include local acidification that negatively modulates OPC function. Moreover, the recruitment of OPCs via proliferation and migration is not always sufficient to repopulate lesions with viable OPCs: approximately 30% of analyzed human lesion samples showed insufficient OPC populations and incomplete remyelination [145]–[147]. Although OPC underpopulation is observed predominantly in chronic lesions, insufficiency of OPCs is also reported for active and remyelinating lesions [147]. Thus, although remyelination can occur at early stages of MS, this process is heterogeneous, often incomplete, and usually results in thinner myelin sheaths compared to primary myelination. Such *in vivo* inefficiency of repair suggests that the lesion microenvironment is suboptimal due to factors that may include acidic pH.

Further, with disease progression and with formation of chronic lesions, the efficiency of both OPC recruitment and differentiation decrease. This results in significantly decreased remyelination [18], as demonstrated for MS lesioned tissue [2], [15], [145]–[147], [157], and for recurring demyelination in animal models [158], [159]. The decline of OPC recruitment and differentiation, and resulting decreased remyelination is also observed with increased age of animals/patients [21], [156], [160]. It is possible that acidic pH is among the negative cues that dominate the chronic lesions and that its negative effect increases with disease progression and aging. In summary, currently insufficient data for spatiotemporal distribution of pH in lesioned tissue obfuscates evaluation of the relative importance of acidic pH for demyelinating lesions *in vivo*. Mapping pH within lesions (and over time) will enable correlation with OPC proliferation, differentiation and remyelination. At present, the findings *in vitro* and the demonstrated differences *in vivo* within and among lesions in OPC recruitment, differentiation, and remyelination extent together suggest that pH could modulate OPC biology and remyelination efficiency *in vivo*.

## Conclusions

The present results demonstrate that acidic extracellular pH, which is characteristic of demyelinating lesions, can affect the major components of OPC biology involved in post-demyelination response. Acidic extracellular pH reduced OPC motility in a manner dependent on ligand-receptor interactions at the cell-surface interface, and decreased OPC proliferation, survival, and differentiation. These *in vitro* findings prompt consideration that the *in vivo* acidic environment of demyelinating lesions may be a factor contributing to the decrease of remyelination extent. Moreover, OPC migration, survival, and proliferation were maximal within a limited range of pH 7.0–7.5, and decreased in both more acidic and more alkaline conditions, indicating that *in vivo* deviation from this permissive pH range may affect each of these processes. Within a pH gradient consistent with that of the interface between demyelinating lesion and healthy CNS tissue, OPCs migrated toward the more acidic region. Although this directional migration may contribute to OPC recruitment toward acidic lesions, the decreased motility, survival, proliferation, and differentiation to myelin-producing oligodendrocytes at pH<6.5 may promote a cumulative negative effect on CNS remyelination.
